# A Decade of Progress Using Virtual Reality for Poststroke Lower Extremity Rehabilitation: Systematic Review of the Intervention Methods

**DOI:** 10.1155/2015/342529

**Published:** 2015-10-11

**Authors:** Carlos Luque-Moreno, Alejandro Ferragut-Garcías, Cleofás Rodríguez-Blanco, Alberto Marcos Heredia-Rizo, Jesús Oliva-Pascual-Vaca, Pawel Kiper, Ángel Oliva-Pascual-Vaca

**Affiliations:** ^1^Department of Physical Therapy, Faculty of Nursing, Physiotherapy and Podiatry, University of Seville, C/Avicena s/n, 41009 Seville, Spain; ^2^Motion Analysis Laboratory, “Virgen del Rocio” Hospital, Physiotherapy Area, Avenida Manuel Siurot, 41013 Seville, Spain; ^3^Laboratory of Kinematics and Robotics, IRCCS San Camillo Hospital Foundation, Via Alberoni 70, 30126 Venice, Italy; ^4^Department of Nursing and Physiotherapy, University of the Balearic Islands, Carretera de Valldemossa, km 7,5, 07122 Palma de Mallorca, Spain; ^5^Department of Physical Therapy, Francisco Maldonado University School of Osuna, Camino de los Cipreses 1, Osuna, 41640 Seville, Spain

## Abstract

*Objective*. To develop a systematic review of the literature, to describe the different virtual reality (VR) interventions and interactive videogames applied to the lower extremity (LE) of stroke patients, and to analyse the results according to the most frequently used outcome measures. *Material and Methods*. An electronic search of randomized trials between January 2004 and January 2014 in different databases (*Medline, Cinahl, Web of Science, PEDro, * and *Cochrane*) was carried out. Several terms (*virtual reality, feedback, stroke, hemiplegia, brain injury, cerebrovascular accident, lower limb, leg*, and *gait*) were combined, and finally 11 articles were included according to the established inclusion and exclusion criteria. *Results*. The reviewed trials showed a high heterogeneity in terms of study design and assessment tools, which makes it difficult to compare and analyze the different types of interventions. However, most of them found a significant improvement on gait speed, balance and motor function, due to VR intervention. *Conclusions*. Although evidence is limited, it suggests that VR intervention (more than 10 sessions) in stroke patients may have a positive impact on balance, and gait recovery. Better results were obtained when a multimodal approach, combining VR and conventional physiotherapy, was used. Flexible software seems to adapt better to patients' requirements, allowing more specific and individual treatments.

## 1. Introduction

Virtual reality (VR) is an innovative technology that describes a scenario generated by a computer (virtual world), in which the users can interact. This allows creating multisensorial stimuli that transfer the complexity of the physical world to another controlled environment, in which it is possible to modify and control a great number of physical variables [1]. The computational approach means an important analysis for the motor system in the neuroscience field, which offers the opportunity to unify experimental data in a theoretical framework. This approach allows the patient to make more precise and effective movements through a sensorimotor feedback [2]. With regard to stroke patients, the loss or impaired ability to walk is one of the most devastating consequences, and gait recovery has been recognized as a primary objective in stroke rehabilitation [3].

For the past decade, there has been a significant progress in the use of VR systems for the recovery of the plegic lower extremity (LE) after stroke, [4–10] with positive results, not only in gait but also in variables such as balance. Apart from specific VR, adaptation of interactive videogames to the stroke patient's rehabilitation provides an interesting and useful approach. Due to the heterogeneity of the different trials in the literature, it is difficult to reach a conclusion concerning the most important aspects to take into account to create an efficient VR system for poststroke lower extremity rehabilitation. The objective of this trial was to make a systematic review of the literature, to update and describe the different VR interventions or interactive videogames that have been used for LE recovery in stroke patients, and to analyze the previous findings according to the most frequently used variables.

## 2. Material and Methods

### 2.1. Identification of Trials

A search of articles published between January 2004 and January 2014 was carried out in different electronic databases (*Medline, Cinahl, Web of Science, PEDro,* and* Cochrane*) by two independent reviewers. This term combination (virtual reality OR feedback) AND (stroke OR hemiplegia OR brain injury OR cerebrovascular accident) AND (lower limb OR leg OR gait) was used to find those sources considered to be relevant.

Reviewers also performed a manual bibliographic search of full texts and reviews, in order to identify additional relevant studies, including congress contributions and cited references.

### 2.2. Eligibility Criteria

Randomized controlled trials were only included if the study design compared pre- and postintervention values. Likewise, the treatment had to be specifically referred to VR techniques or interactive videogames used for the LE recovery in stroke patients rehabilitation, either compared to an alternative intervention or not. The studies were considered for review purposes only if patients had a single stroke episode, with no restrictions of mean age of length or recovery. On the contrary, case series studies, single case clinical reports, and review studies were not included for assessment in the present systematic review. Likewise, studies with a sample of hemiplegic subjects after a medical diagnosis different from ischemic or hemorrhagic stroke were excluded.

After a first selection process, only those trials, in which proper assessment scales (with validity and reliability) were used, were finally included.

The* PEDro* scale was used to analyse the methodological quality of each trial by two independent reviewers [11].

**Figure 1:**
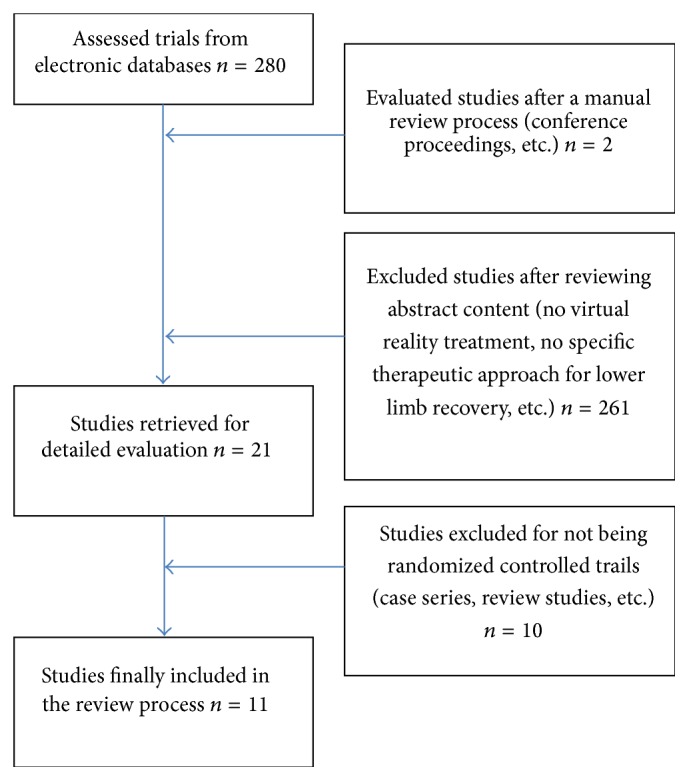
Flow chart of the articles selection.

Figure 1 shows the flow chart diagram of the study selection process.

## 3. Results

After the selection process, 11 randomized controlled trials (RCT) were included for assessment purposes [3, 12–21], accounting for a total of 9 different projects since two groups of authors [14, 16, 18, 21] published study data from the same trial in different articles. All the included trials account for a total of 183 participants (Table 1). To the best of authors' knowledge, there are no RCT on this matter before 2004. Most trials have shown moderate to high quality, with total scores ranging from 6 to 10 in the* PEDro* scale. Table 1 lists the specific scores of each study. Table 1 also includes the number of patients in each study, as well as aspects related to the mean age of the study sample, the time since the stroke onset (taken six months as a reference to differentiate subacute poststroke patients from chronic poststroke subjects), the treatment approach used in the trial, the number and average of treatment sessions, and the main outcome measures.

The present findings show that sample sizes were generally quite small in all studies, with a mean sample of around 20 subjects, equally distributed into the intervention or the control group. The mean age of the participants ranged between 47 and 66 years. The youngest study samples were those from Gil-Gómez et al. [17] and Park et al. [20] with a mean age below 50 years (Table 1).

All patients can be considered to be in a chronic poststroke stage (more than 6 months after the stroke episode). However, even though they were all in a chronic stage, there is a great heterogeneity between studies with regard to the time of recovery after the stroke and the baseline study assessment, which makes it difficult to compare between trials. While studies from You et al. [13], Gil-Gómez et al. [17], Cho and Lee [19], and Cho and Lee [21] included patients with a 1- to 2-year mean period of time after the stroke, Park et al. [20] evaluated individuals with more than 10 years of poststroke recovery.

The total number and week average of treatment sessions has been also taken into account when comparing the different studies. The number of sessions varies between 6 and 20, with a mean average of 15 sessions.

According to the eligibility criteria, the study design of all trials was based on a comparison of postintervention and preintervention scores after the VR treatment. In most cases, the VR treatment lasted one hour per day. However, the treatment duration was inferior in some trials, between 20 minutes and one hour, with a mean frequency of 3 times per week. The VR intervention approach was compared to either conventional physiotherapy, an alternative intervention, or no treatment. In some trials, the impact of a multimodal approach combining VR and conventional physiotherapy was assessed.

As previously mentioned, there are different types of virtual environments, according to their level of immersion:

- nonimmersive VR, in which the computer generates environments that are projected either on a screen or on a wall in front of the patient;
- semi-immersive VR or augmented reality, which overlays virtual images to real images increasing the informative content of the real ones;
- immersive VR, in which the viewer is a part of the environment. One example is head mounted display (HMD), a device with a helmet that provides images within a computer, as a unique visual stimulation [1].

All the assessed trials used different types of virtual environments. Immersive systems are purported to be more effective because they provide a more intense feeling of reality; however, they may provoke “cybersickness” (symptoms such as vomiting, dizziness) in some participants [22]. For this reason, most authors prefer to choose semi-immersive systems.

The visual/auditory feedback was used in most of the studies, along with the knowledge of results/performance (KR/KP) approach. On the other hand, the vibrotactile feedback was not frequently used.

We will briefly describe the VR systems that were used in each trial.

(i) Jaffe et al. [12] compared two training groups: real and virtual. In the first one, real obstacles were used whereas in the virtual group a head-mounted device was used to observe the simultaneous registration of the legs' real movement, introducing virtual stationary images of obstacles and getting a patient's feedback. The virtual obstacle training generated greater improvements in gait velocity compared with real training during the fast walk test and the self-selected walk test. Overall, subjects showed clinically meaningful changes in gait velocity, stride length, walking endurance, and obstacle clearance capacity as a result of either training method.

(ii) You et al. [13] compared a control group (no intervention) with a group that used the IREX VR system, which allows interaction (within cybergloves, etc.) with virtual objects in environments that can be individualized. This allows optimizing motor relearning. Three specific exercises were included: going up/down stairs, diving among sharks, and snowboarding. VR seems to induce cortical reorganization from aberrant ipsilateral to contralateral primary sensorimotor cortex activation. In this study, motor function was significantly improved after VR.

(iii) Mirelman et al. [14, 16] compared a training treatment based on a robotized system of VR (Rutgers Ankle) with another intervention only based on a robot. The Rutgers Ankle system consists of a Stewart platform with six-degree feedback strength of foot freedom and a screen that allows the patient to train the LE. While the patient is sitting and simulates driving a boat or a plane, parameters are individualized by the physiotherapist. The LE training that combined a robotic device and VR improved walking ability in the laboratory and the community (velocity and distance walked) and showed a higher impact than the robot training alone. The main observed effects after training included improved motor control at the ankle, which enabled the patient to do other functional improvements.

(iv) Yang et al. [3] compared training on a treadmill with training in a VR system composed of a treadmill, a screen with a high vision field, and a 3D capture system (Fastrak Polhemus) for leg movements. Virtual scenarios represented a field, some obstacles, and a task to cross a street. Gait speed was increased in each session. VR group improved significantly more than treadmill group in walking speed and community walking time in a short term and in Walking Ability Questionnaire score at a follow-up period.

(v) Kim et al. [15] measured the additional effect of the VR system to a conventional physiotherapy approach. The same system as previously described by You et al. [13] was used. The VR group improved BBS scores, balance, and dynamic balance angles (ability to control weight shifting) compared to the patients that underwent only physiotherapy.

(vi) Gil-Gómez et al. [17] compared an intervention program with the* Nintendo Wii Balance Board* (WBB) with eBaViR to a conventional physiotherapy treatment in patients with brain damage. Although 6 of the 17 patients had hemiplegia not secondary to a stroke, this trial was also included for the treatment approach that it shows since flexible software for* Wii*, specific for rehabilitation, was designed. Patients using WBB had a significant improvement in static balance (BBS and ART), compared to patients who underwent traditional therapy. With regard to dynamic balance, there were no differences between study groups.

(vii) Fritz et al. [18] compared an experimental group that used* Nintendo Wii* (*Wii Sports* and* Wii Fit*) and* Play Station* (*Eye Toy Play 2* and* Kinetic*) with a control group that underwent no intervention. No statistically significant differences in the comparison between or within groups were found, either in the short term or in the follow-up process.

(viii) Cho and Lee [19, 21] also combined VR and conventional physiotherapy. One group of patients underwent a training treatment on a treadmill and another group used the* TRWVR system* (*treadmill training based real*-*world video recording*), which uses different virtual environments registered in the real world (paths, obstacles, etc.). The TRWVR group showed better results in walking balance (BBS and TUG), dynamic balance gait, and spatiotemporal parameters (velocity and cadence) than the treadmill group.

(ix) Park et al. [20] compared a VR approach with a conventional physiotherapy treatment. They used a programme for improving postural control and gait ability through a visual feedback, comparing the reference scenario of the movement and the real movement. In the gait parameters, subjects in the VR group showed a significant improvement, except for cadence immediately after training and at the follow-up when compared to the conventional physiotherapy group. In the comparisons between groups, the VR group also showed significantly greater improvement only in stride length compared with the control group. On the contrary, no significant differences were found in other gait parameters.

## 4. Discussion

The validity and reliability of the outcome measures used in the different trials are crucial to determine the quality of the findings. The most frequently used outcome measures were gait speed, balance, and improvement of the motor function.

### 4.1. Gait Speed

Gait speed was used as an outcome assessment tool in 8 of the 9 selected trials. On the other hand, You et al. [13] evaluated motor function, functionality, and cortical changes by means of nuclear magnetic resonance.

Gait speed is considered to be a significant, sensitive, and reliable tool to evaluate the impairment severity and the community's functional ability to walk [9].

The* 6-Minute Walking Test* has been the most frequently used test to measure gait speed. Systems of movement analysis to measure space-time parameters and the* 10-Meter Walking Test* were also used.

Most of the trials evaluated spontaneous gait speed. However, some authors (Jaffe et al. [12] and Fritz et al. [18]), who observed a significant increase of maximum speed in the VR group but no significant changes in spontaneous speed, considered both spontaneous and maximum speeds. A plausible reason may be the small number of treatment sessions [12] and the lack of physiotherapist's guidance [18] during the intervention.

In the rest of the trials, there was a significant difference in the spontaneous gait speed in the VR group compared to the control group, except for Gil-Gómez et al. [17]. A possible reason is that the intervention aimed to improve balance, but exercises were not oriented for improving speed. Park et al. [20] designed an intervention program focused on improving posture. In addition, poststroke time of recovery in both groups was quite long and, therefore, we may assume less neuronal plasticity in the study sample.

### 4.2. Balance

Balance was used as an outcome measure in 4 of the 9 selected trials. Mirelman et al. [14, 16] only used the* Berg Balance Scale* (BBS) in the baseline assessment but not in the postintervention evaluation. Jaffe et al. [12] also employed a balance test to describe the initial stage. Although some authors used additional scales, the BBS was used in all of them.

Three out of 4 trials that compared postintervention balance scores with baseline assessment concluded significant improvement results in the VR group for balance as measured by means of the BBS. Fritz et al. [18] did not find any significant differences in their first pilot study. Since they used two different videogames, it is difficult to state the specific influence of each system on the results. However, as stated by them, a physiotherapist's guidance is necessary to facilitate and orientate the patient about the most suitable motor strategies in order to find better and clinically significant results [6, 18].

Gil-Gómez et al. [17] observed significant differences in balance, by comparing an intervention group with the* Wii Balance Board* (WBB) and a control group with a conventional physiotherapy approach. It must be pointed out that, in the WBB group, the software was flexible, and the physiotherapist was constantly adjusting the degree of difficulty and other parameters. This issue may demonstrate the importance of physiotherapist's intervention in this type of systems, since results are poorer when the patient is not guided [18].

### 4.3. Motor Function of the Plegic LE

Improvement of motor function was evaluated in 3 trials. You et al. [13] and Kim et al. [15] used the MMAS (*Modified Motor Assessment Scale*). On the contrary, Fritz et al. [18] used the* Fugl-Meyer* (FM) scale, in which it is specific for LE assessment. Only the first two studies obtained significantly better results in the VR group.

Other authors performed measurements of space-time and kinematic-kinetic parameters within systems of gait analysis [12, 15, 16, 20, 21]. They all obtained a significant improvement in the VR group, except for Park et al. [20]. A plausible reason is that the time of recovery after the stroke was too long in the latter study.

In recent years, the use of VR systems for the functional recovery of the gait poststroke has increased. Despite the fact that some systematic reviews have been published in the issue, most of them are focused on the motor recovery of the upper limb [23]. Therefore, it is important to build stronger evidence about the impact of VR treatment on LE recovery, because only 4 articles [24, 25] on this issue have been formerly reviewed.

As a main finding, it seems that patients should receive a minimum number of ten sessions for the intervention to be effective. In most cases, better results were obtained when a multimodal approach combining VR and conventional physiotherapy was used. However, a more homogeneous methodology is crucial and future research is needed to elucidate the possible effect of each individual system in gait and LE recovery after stroke. It is also important to carry out a proper baseline assessment to establish which system is the most appropriate for handling the different disorders (balance, functionality, gait speed, etc.). An early treatment after stroke is also important [26], because intervention seems to be less effective when the recovery time after stroke is too long. Neural plasticity may be a factor to understand this aspect.

## 5. Conclusions

The number of trials is small and the evidence is limited. Nevertheless, the present findings seem to suggest that VR intervention has a positive impact on balance and gait after stroke.

The use of commercial videogames in rehabilitation seems to be useful. However, a proper physiotherapist's guidance is needed to facilitate and orientate the patient on the most suitable motor strategies. It is also necessary to develop more flexible software to individualize treatments and adapt the intervention to the patients requirements.

## Figures and Tables

**Table 1:** Synthesis of results.

Author year *PEDro *	Group/sample	Age range years	Time since stroke onset	Type of VR	Type of feedback	Sessions	Outcome measures

Jaffe et al. (2004) [12] *PEDro*: 4/10	ROT: 10 OVR: 10	60 mean	Chronic 3-4 years	Immersive: VR scripting language	Visual, vibrotactile, auditory	6 ses. 1 h/d, 3 d/w, 2 w	Stride gait analysis system, obstacle test, 6MWT

You et al. (2005) [13] *PEDro*: 5/10	VR: 5 C: 5	54 mean	Chronic 1-2 years	Semi-immersive: IREX VR system	KR/KP	20 ses. 1 h/d, 5 d/w, 4 w	FAC, MMAS, fMRI

Mirelman et al. (2009) [14] *PEDro*: 5/10	R + VR: 9 R: 9	61 mean	Chronic 3-4 years	Semi-immersive: Rutgers Ankle	KR/KP	12 ses. 1 h/d, 3 d/w, 4 w	Gait speed, 6MWT, PAM (gait spatiotemporal parameters)

Yang et al. (2008) [3] *PEDro*: 6/10	TT: 9 VRTT: 11	58 mean	Chronic 6 years	Immersive: visual screen, 3D Fastrak Polhemus	Auditory, visual	9 ses. 20 m/d, 3 d/w, 3 w	Walking speed, WAQ, ABC scale

Kim et al. (2009) [15] *PEDro*: 6/10	CP + VR: 12 CP: 12	51 mean	Chronic 2-3 years	Semi-immersive: IREX VR system	KR/KP	16 ses. CP: 40 m/d, 4 d/w, 4 w VR: 30 m	BPM, BBS, 10MWT, MMAS, GAITRite

Mirelman et al. (2010) [16] PEDro: 3/10	R + VR: 9 R: 9	61 mean	Chronic 3-4 years	Semi-immersive: Rutgers Ankle	KR/KP	12 ses. 1 h/d, 3 d/w, 4 w	Gait analysis (kinematic and kinetic parameters)

Gil-Gómez et al. (2011) [17] *PEDro*: 6/10	WBB: 9 CR: 8	47 mean	Chronic 1-2 years	Nonimmersive: WBB easy balance virtual rehabilitation	Auditory, visual	20 ses. 1 h/d (3–5 d/w)	BBS, BBA, ART, TST, ST, 1MWT, 10MWT, TUG, 30SST

Fritz et al. (2013) [18] *PEDro*: 6/10	Game: 15 C: 13	66 mean	Chronic 3-4 years	Nonimmersive and semi-immersive: WBB and play station	Auditory, visual	20 ses. 1 h/d, 4 d/w	FMS, BBS, TUG, 6MWT, Dynamic Gait Index, 3MWT, Stroke Impact Scale

Cho and Lee (2013) [19] *PEDro*: 7/10	CP + TRWVR: 15 CP + TT: 15	64 mean	Chronic 1-2 years	Semi-immersive: treadmill + screenshot of real-world video recording	Auditory, visual	18 ses. 30 m/d, 3 d/w, 6 w	BBS, TUG, gait performance (GAITRite)

Park et al. (2013) [20] PEDro: 4/10	VR + CP: 8 CP: 8	47 mean	Chronic 11 years	Immersive VR-based postural control program (head-mounted display)	Visual	12 ses. 30 min/d, 3 d/w, 4 w	10MWT, GAITRite (gait parameters)

Cho and Lee (2014) [21] *PEDro*: 7/10	CP + TRWVR: 15 CP + TT: 15	64 mean	Chronic 1-2 years	Semi-immersive: treadmill + screenshot of real-world video recording	Auditory, visual	18 ses. 30 m/d, 3 d/w, 6 w	BBS, TUG, Postural Sway Platform System, GAITRite

3MWT: 3-Minute Walking Test; 6MWT: 6-Minute Walking Test; 10MWT: 10-Meter Walking Test; 30SST: 30-Second Sit to Stand Test; ABC scale: activities-specific balance confidence; ART: Anterior Reach Test; BBA: Brunel Balance Assessment; BBS: Berg Balance Scale; BPM: Balance Performance Monitor; C: control (no intervention); CP: conventional physiotherapy; FAC: Functional Ambulatory Scale; FMS: Fugl-Meyer Scale; KP: knowledge of performance; KR: knowledge of results; MMAS: Modified Motor Assessment Scale; OVR: virtual obstacle training; PAM: Patient Activity Monitor, Ossur (walking activity); R: robot; ROT: real obstacles training; ST: Steeping Test; TRWVR: treadmill training based real-world video recording; TST: Timed Stair Test; TT: treadmill training; TUG: Timed Up Go; VRTT: virtual reality-based treadmill training; WAQ: Walking Ability Questionnaire; WBB: *Wii Balance*.
